# Drug Usage Evaluation of Dapsone

**DOI:** 10.4103/0250-474X.57301

**Published:** 2009

**Authors:** G. Kannan, J. Vasantha, N. Vanitha Rani, P. Thennarasu, K. Kousalya, P. Anuradha, C. Umamaheswara Reddy

**Affiliations:** Department of Pharmacy Practice, Sri Ramachandra College of Pharmacy, Sri Ramachandra University, Porur, Chennai-600 116, India

**Keywords:** Dapsone, drug usage evaluation, adverse effects, patient education

## Abstract

Dapsone has been the principal drug in a multidrug regimen recommended by the World Health Organization for the treatment of leprosy. It is also widely used by dermatologists in varied skin conditions like dermatitis herpetiformis, bullous pemphigoid, Behcet's disease, lupus erythematous and a host of other skin diseases. Hence an attempt has been made to review the utilization and qualitative evaluation of dapsone over a period of 6 months in a tertiary care teaching hospital. The study consisted of 80 patients (54 leprosy and 26 non-leprosy patients), prescribed with dapsone 100 mg oral once daily. The prescribing patterns of dapsone in leprosy and other dermatological conditions (non-leprosy) were analyzed and the safety, efficacy and appropriateness of the doses prescribed were reviewed. The adverse drug reactions observed in the study population were type I Lepra reactions, gastrointestinal side effects (abdominal pain and anorexia), peripheral neuropathy, other nervous side effects (insomnia, headache and vertigo) and other adverse reactions (fever and tinnitus). Patient information leaflets were distributed to patients to educate on the appropriate use of dapsone.


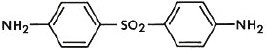


Dapsone (4,4'-diaminodiphenylsulfone) was synthesized a century ago (1908) and continues to be a powerful therapeutic tool in many skin diseases[1]. It has an antibacterial spectrum and a similar mechanism of action as sulphonamide. It is the drug of choice in chemotherapy of leprosy. Other analogs owe their activity and toxicity to dapsone released from them. Dapsone is therefore the preferred sulfone being cheaper than and as effective as others. It is well absorbed by the oral route, preferably at lower doses and acetylated in the liver prior to elimination. It has been found that there are slow and fast acetylators among the patient population. This variability does not affect the clinical utilization of dapsone. The drug is also N-hydroxylated in the liver which product is responsible for the hemodynamic adverse effects. Its half life is long, 10-50 h and the time to reach plateau is at least 8 days[2]. The drug has been used for other skin disorders also. It has been reported for use in acne but less effective than retinoic acid[3]; for cutaneous manifestations of Behcet's disease requiring further trials[4]; for the treatment of bullous pemphygoid as an adjunct therapy[5]; combined with trimethoprim it is as effective as co-trimoxazole in *Pneumocystis carinii* pneumonia[6]; it is a useful suppressant in treating dermatitis herpetiformis[7] and relapsing Polychondritis[8]; for vesico bullous lesions of lupus erythematous, it has been recommended as first line systemic therapy[9]; its use has been reported in pemphigus herpetiformis[10], pyoderma gangrenosum[11]; dapsone reduces the local and systemic reactions to spider bite[12] and found to be useful for urticarial vasculitis syndrome[13].

A variety of adverse effects have been recognized with dapsone therapy. The most frequent and well-documented adverse drug reactions (ADRs) being methemoglobinemia (caused by the hydroxylamine metabolite), agranulocytosis, hypersensitivity syndrome, psychosis and neuropathy. Some useful drug interactions have been documented with fluconazole which reduce the adverse reactions to dapsone by reducing the production of its toxic metabolite and with cimetidine which reduces methemoglobinemia due to dapsone. Of an adverse drug interaction, didanosine is known to cause increased peripheral neuropathy when given with dapsone. Dapsone-induced methemoglobinemia is treated with 1-2 mg/kg methylene blue by slow intravenous drip in emergency situation or orally 3-5 mg/kg every 4-6 h in non-emergency[14].

This study was initiated to understand the prescribing patterns of dapsone, its safety, efficacy, and to educate patients through information leaflets on the proper use of dapsone thereby emphasizing the primary responsibilities a clinical pharmacist could share with the health care team.

The Institutional Medical Ethics Committee permission was obtained before performing the study. Patients visiting the out-patient skin clinics of Sri Ramachandra Hospital were enrolled for this study. The dermatology department has provided special clinics every day; Monday-vitiligo, Tuesday-psoriasis, Wednesday-leprosy, Thursday-eczema, Friday-acne and autoimmune and bullous disorders every Saturday. Patients who attended the leprosy clinic on Wednesdays and autoimmune disorders on Saturdays and who were prescribed dapsone by the dermatologists were taken for the study. During the study period, 80 patients were reviewed. Of the 80, only 19 (7 leprosy and 12 non-leprosy) were newly recruited and the remaining 61 patients (47 leprosy and 14 non-leprosy) were on regular follow-up. A patient data collection form was prepared and the data on demographic details (name, age, sex), past medical history, social history, biochemical values such as hematological, liver function tests, renal function tests and relevant histopathological findings were procured and analyzed. Patients were counseled after their consultation with the dermatologists regarding the dose, dosing interval, side effects, adverse drug reactions and their follow-up schedule to the dermatology clinic. Patient information leaflets on dapsone were prepared in English (Appendix 1) and the vernacular language (not shown) and these were given to all patients after oral counseling. They were also educated to report to their physician/pharmacist, in case of any side effects experienced by them.

The patients were classified as leprosy (54) and non-leprosy (26) patients. The 54 leprosy patients (33 males, 21 females; mean age 40.48±14.67 years) were categorized by the dermatologists as shown in (Table 1). These patients were prescribed dapsone 100 mg orally O.D. for a period of 6 months. In addition to dapsone, 47 patients were administered rifampin 600 mg as a single dose once a month for 6 months, one patient with rifampin 450 mg and one patient with rifampin 300 mg single dose once a month for 6 months; 10 patients were prescribed with clofazimine 100 mg O.D. orally for 6 months, one patient with clofazimine 50 mg O.D. per oral for 6 months; 14 patients were prescribed with oral prednisolone as follows: 2 patients were given 5 mg, 3 patients 10 mg, 2 patients 20 mg, 3 patients 25 mg and 4 patients 30 mg O.D. for 6 months.

**TABLE 1:** CLASSIFICATION OF LEPROSY PATIENTS

Type of Leprosy	No of Patients (n=54)	
	
	n	%
Paucibacillary	41	75
Multibacillary	10	19
Neuritic	3	6

Majority of the patients had paucibacillary leprosy.

Twenty six non-leprosy patients (14 males and 12 females; mean age 38.15±15.20 years) were diagnosed by the dermatologists as having other dermatological conditions (explained in Appendix 2) as listed in (Table 2). These patients were prescribed dapsone 100 mg O.D. In addition to dapsone, 16 patients were prescribed oral prednisolone as shown: 4 patients- 5 mg O.D., 3 patients-10 mg O.D., 2 patients- 15 mg O.D., 4 patients-20 mg O.D. and 25 mg O.D. for 3 patients.

**TABLE 2:** CLASSIFICATION OF NON-LEPROSY PATIENTS

Type of Diseases	No of Patients (n=26)	
	
	n	%
*Lichen planus*	16	62
*Bullous pemphygoid*	2	7
*Pemphigus vulgaris*	2	7
Acne	1	4
*Pemphigus erythematosus*	1	4
Familial benign pemphigus	1	4
*Dermatitis herpetiformis*	1	4
Bullous lichen planus irritant dermatitis	1	4
*Psoriasiform dermatitis*	1	4

Skin conditions for which dapsone had been used in non-leprosy patients, of which, lichen planus was predominant.

Of the 54 leprosy patients, only one patient was a known diabetic and one known hypertensive. In 24 non-leprosy patients, one had hypertension, 2 had diabetes and 3 had dermatitis. The social history of all patients show that 3 were cigarette smokers, 2 on tobacco chewing and one was a heroin drug abuser. Biochemical parameters were normal for all the patients during the 6 month study period, but the past medical records of 47 patients who were on follow-up had an elevated eosinophil documented in 3 patients only. Adverse reaction events of the 80 patients were recorded in both groups of leprosy and non-leprosy patients. Peripheral neuropathy was the ADR of dapsone reported by majority of the leprosy patients (35%) and 18% of the non-leprosy patients. Gastrointestinal effects (abdominal pain and anorexia) were reported by 25% of leprosy group and 55% of non-leprosy group. Other nervous effects included insomnia; headache and vertigo were seen in 20% of the leprosy group and 27% of the non-leprosy group. Type I lepra reactions (delayed hypersensitivity reactions caused by increased recognition of *Mycobacterium leprae* antigens in skin and nerve sites in borderline tuberculosis patients) and other ADRs such as fever and tinnitus were reported by each 10% leprosy patients only (Table 3). All the patients were counseled orally at the time of their hospital visit and patient information leaflets on dapsone were provided to all patients at the time of counseling.

**TABLE 3:** ADVERSE DRUG REACTIONS IN STUDY POPULATION

ADRs	Leprosy patients (n = 54)		Non-leprosy patients (n=26)	
		
	N	%	n	%
Lepra reactions-type I	2	10	0	0
Peripheral Neuropathy	7	35	2	18
Other nervous effects	4	20	3	27
Gastrointestinal effects	5	25	6	55
Other ADRs	2	10	0	0

Majority of the leprosy patients have reported of peripheral neuropathy where as gastrointestinal side effects were more common among the non-leprosy group.

Dapsone was one of the chemicals reviewed by the Royal Commission, which recommended evaluation of carcinogenicity of dapsone as it was used during the Vietnam conflict by the Australian forces for the treatment of falciparum malaria. The study did not reveal any evidence of cancer incidence with dapsone exposure[15].

Dapsone treatment has been found to be effective with no serious hematological complications in both leprosy and non-leprosy patients. No serious laboratory abnormalities were noted as only 3 patients have reported elevated eosinophil count. In this study, the adverse effects reported by the study group were not serious and were managed symptomatically. Dapsone was not withdrawn for any of the patients as none of them reported of any severe adverse side effects. None of the patients have reported dapsone hypersensitivity syndrome which is a rare hypersensitivity reaction to dapsone. Therefore dapsone is a beneficial, safe and inexpensive therapy in both leprosy and non leprosy patients.

Patient education provided to the patient improved patient compliance as all the patients were regular for their follow-up and reported the side effects, if any occurred to the physician /pharmacist during their follow-up visit. This helped the physician to be aware of the side effects experienced by the patients and to avoid the incidence of any serious ADR.

Dapsone is available as a topical gel in the US to be used only with the doctor's prescription. It is used for the treatment of acne. The same precautions for dapsone tablets may be taken when dapsone is used as topical preparation. In this study, the clinical pharmacist had a leading role in addressing the use, effectiveness, adverse drug reactions, drug interactions commonly seen with dapsone. The fears arising out of adverse drug reactions of dapsone was allayed through effective counseling and distributing patient information leaflets to motivate patient compliance.

## APPENDIX 1: PATIENT INFORMATION LEAFLET ON DAPSONE[16]

What is Dapsone?

Dapsone is an anti-infective medication.

Dapsone is used to treat leprosy and skin infections.

What should I discuss with my doctor before taking dapsone?

Tell your doctor if you are allergic to dapsone or other sulpha drugs.

Do not take dapsone without first talking to doctor if you are pregnant, planning for pregnancy, or are breast feeding.

Inform your doctor if you are anemic, have liver or kidney disease or undergoing a surgery within two months including dental surgery.

Tell your doctor if you are taking other medicines, including those available to buy without a prescription, herbal or complementary medicines.

How should I take dapsone?

Take tablet once a day or as directed by the physician.

Try to take dapsone at the same time each day to avoid missing any doses.

Take each dose with a full glass of water.

Taking dapsone with food may decrease stomach upset, should it occur.

It is important to take dapsone regularly to get the most benefit.

Follow all appointments with your doctor regularly. Your doctor may want you to have blood tests or other forms of monitoring during treatment with dapsone.

**What happens if I miss a dose?**

If you forget to take a dose, take one as soon as you remember unless it is nearly time for your next dose. Do not take two doses at the same time to make up. If in doubt, speak to your doctor.

**What should I avoid while taking dapsone?**

Avoid alcohol.

Do not take any other medicines during treatment with dapsone without your doctor's consent.

Avoid prolonged exposure to sunlight. Dapsone may increase the sensitivity of the skin to sunlight and sunburn may be more likely to occur.

If exposure to the sun is unavoidable, wear a sunscreen and protective clothing.

Use caution while driving or performing other tasks requiring mental alertness as dapsone may cause blurred vision or dizziness.

**What are the possible side effects of dapsone?**

Notify your doctor if you experience fever, muscle weakness, paleness, purple discoloration of skin, skin rash, sore throat, yellowing of skin or eyes. If you experience nerve pain or pricking, stabbing pain in legs or feet, soak your feet in cold water for 15-20 minutes and use softsoled, loose fitting shoes with thick socks.

**What other drugs will affect dapsone?**

Many other drugs can interact with dapsone, especially those that may also affect the blood. Do not take any other prescription or overthe-counter medicines, including vitamins, minerals, and herbal products, during treatment with dapsone without first talking to your doctor.

**How to store dapsone tablets?**

Store the medicine away from heat and direct sunlight. Do not store in bathroom, kitchen sink or any damp place. Be sure to discard the outdated medicine.

Keep out of reach of children. Do not let anyone to use your medicine.

## APPENDIX 2: DERMATOLOGICAL CONDITIONS OF THE NON-LEPROSY PATIENTS[17,18]

**Table:**

DISEASE	DEFINITION
1. *Lichen planus*	An inflammatory papulosquamous disorder, characterized by the formation of flat, topped, polygonal, grayish white, purple or lilac eruptions.
2. *Bullous pemphigoid*	An autoimmune skin disorder producing chronic, pruritic bullous eruptions in elderly patients with occasional mucous membrane involvement.
3. *Pemphigus vulgaris*	An uncommon potentially fatal, autoimmune disease characterized by intraepidermal bullae and extensive erosions on apparently healthy skin and mucous membranes.
4. Acne	A follicular disorder affecting susceptible pilosebacious follicles, primarily of the face, neck, and upper trunk and characterized by both inflammatory and non inflammatory lesions.
5. *Pemphigus erythematosus*	A chronic condition characterized by appearance of flaccid bullae which arise over an erythematous skin. The lesions are usually limited to the face and chest.
6. *Familial benign pemphigus*	Hereditary recurrent vesiculobullous dermatitis, usually involving the axillae, groin and neck, with crops of lesions that regress over several weeks or months.
7. *Dermatitis herpetiformis*	A chronic eruption characterized by clusters of intensely pruritic vesicles, papules and urticaria like lesions. The cause is autoimmune.
8. Bullous L.P. irritant dermatitis	A condition of Vesicles and bullae on typical lesions of Lichen Planus due to severe basal cell degeneration induced by the inflammatory process
9. *Psoriasiform dermatitis*	A common chronic, inflammatory, persistent or relapsing, scaling skin condition.
